# Root Microbiome Modulates Plant Growth Promotion Induced by Low Doses of Glyphosate

**DOI:** 10.1128/mSphere.00484-20

**Published:** 2020-08-12

**Authors:** Dario X. Ramirez-Villacis, Omri M. Finkel, Isai Salas-González, Connor R. Fitzpatrick, Jeffery L. Dangl, Corbin D. Jones, Antonio Leon-Reyes

**Affiliations:** a Laboratorio de Biotecnología Agrícola y de Alimentos-Ingeniería en Agronomía, Universidad San Francisco de Quito USFQ, Quito, Ecuador; b Instituto de Microbiologia, Universidad San Francisco de Quito USFQ, Quito, Ecuador; c Department of Biology, University of North Carolina at Chapel Hill, Chapel Hill, North Carolina, USA; d Howard Hughes Medical Institute, University of North Carolina at Chapel Hill, Chapel Hill, North Carolina, USA; e Curriculum in Bioinformatics and Computational Biology, University of North Carolina at Chapel Hill, Chapel Hill, North Carolina, USA; f Galapagos Science Center, USFQ-UNC, San Cristobal, Galápagos, Ecuador; Karlsruhe Institute of Technology (KIT)

**Keywords:** glyphosate, hormesis, microbiome

## Abstract

Glyphosate is a commonly used herbicide with a broad action spectrum. However, at sublethal doses, glyphosate can induce plant growth, a phenomenon known as hormesis. Most glyphosate hormesis studies have been performed under microbe-free or reduced-microbial-diversity conditions; only a few were performed in open systems or agricultural fields, which include a higher diversity of soil microorganisms. Here, we investigated how microbes affect the hormesis induced by low doses of glyphosate. To this end, we used Arabidopsis thaliana and a well-characterized synthetic bacterial community of 185 strains (SynCom) that mimics the root-associated microbiome of *Arabidopsis*. We found that a dose of 3.6 × 10^−6^ g acid equivalent/liter (low dose of glyphosate, or LDG) produced an ∼14% increase in the shoot dry weight (i.e., hormesis) of uninoculated plants. Unexpectedly, in plants inoculated with the SynCom, LDG reduced shoot dry weight by ∼17%. We found that LDG enriched two *Firmicutes* and two *Burkholderia* strains in the roots. These specific strains are known to act as root growth inhibitors (RGI) in monoassociation assays. We tested the link between RGI and shoot dry weight reduction in LDG by assembling a new synthetic community lacking RGI strains. Dropping RGI strains out of the community restored growth induction by LDG. Finally, we showed that individual RGI strains from a few specific phyla were sufficient to switch the response to LDG from growth promotion to growth inhibition. Our results indicate that glyphosate hormesis was completely dependent on the root microbiome composition, specifically on the presence of root growth inhibitor strains.

**IMPORTANCE** Since the introduction of glyphosate-resistant crops, glyphosate has become the most common and widely used herbicide around the world. Due to its intensive use and ability to bind to soil particles, it can be found at low concentrations in the environment. The effect of these remnants of glyphosate in plants has not been broadly studied; however, glyphosate 1,000 to 100,000 times less concentrated than the recommended field dose promoted growth in several species in laboratory and greenhouse experiments. However, this effect is rarely observed in agricultural fields, where complex communities of microbes have a central role in the way plants respond to external cues. Our study reveals how root-associated bacteria modulate the responses of *Arabidopsis* to low doses of glyphosate, shifting between growth promotion and growth inhibition.

## Abstract (precis)

Since the introduction of glyphosate-resistant crops, glyphosate has become the most common and widely used herbicide around the world. Due to its intensive use and ability to bind to soil particles, it can be found at low concentrations in the environment. The effect of these remnants of glyphosate in plants has not been broadly studied; however, glyphosate 1,000 to 100,000 times less concentrated than the recommended field dose promoted growth in several species in laboratory and greenhouse experiments. However, this effect is rarely observed in agricultural fields, where complex communities of microbes have a central role in the way plants respond to external cues. Our study reveals how root-associated bacteria modulate the responses of *Arabidopsis* to low doses of glyphosate, shifting between growth promotion and growth inhibition.

## INTRODUCTION

Glyphosate is a commonly used herbicide that inhibits the production of aromatic amino acids by binding reversibly to the 5-enolpyruvylshikimate-3-phosphate synthase (EPSPS) in the shikimate pathway (1). Because the enzyme EPSPS is essential in all higher plants, glyphosate has a broad action spectrum as a herbicide, and its use has increased since the development of glyphosate-resistant commercial crops (i.e., “Round-Up Ready”) (1, 2). Glyphosate at sublethal doses, however, has been shown to induce plant growth, increasing plant dry mass by as much as 125% (2, 3).

This phenomenon, termed hormesis (2), has been reported in some species but is not widespread, suggesting that hormesis depends on multiple factors that are not yet well understood. For instance, most of these studies were performed under microbe-free conditions or under conditions with a reduced microbial load by using sterilized substrates in open or closed systems (3–7). Only a few examples were performed in agricultural fields (8, 9), where the microbial community is intact (10). The application of standard doses of glyphosate is known to produce small changes in the soil microbiome composition (1, 11). Glyphosate also alters the gene expression of bacteria in the rhizosphere, reducing carbohydrate and amino acid metabolism transcripts and enhancing protein metabolism, respiration, and gene transcription (11).

As a result of its effects on rhizosphere microbes, we hypothesized that the root microbiome modulated the growth promotion induced by glyphosate at low doses. To test this idea, we used Arabidopsis thaliana as a plant model and a synthetic bacterial community (SynCom), previously characterized in other studies (12, 13), as a model root microbiota. The SynCom was composed of 185 bacterial isolates from the root endophytic compartment of healthy *Arabidopsis* plants grown in two wild soils (12). The main advantage of this system is that it allows us to contrast a bacterial community representative of a natural root microbiome with an uninoculated control under identical environmental conditions (12, 14).

## RESULTS

### Shoot growth promotion induced by low doses of glyphosate is lost in the presence of SynCom.

We established the glyphosate dose by testing a thousandfold and a millionfold dilution of the recommended glyphosate dose (3.6 g acid equivalent [a.e.]/liter) on uninoculated *Arabidopsis* plants. We selected 3.6 × 10^−6^ g a.e./liter as a low dose of glyphosate (LDG), since plants grown on this treatment had an increase in the shoot dry weight (see Fig. S1 in the supplemental material).

10.1128/mSphere.00484-20.1 FIG S1 Glyphosate dose standardization. Seven-day-old seedlings were transferred to half-strength MS medium without glyphosate (0.0) or amendment with 3.6 × 10^−6^ and 3.6 × 10^−3^ g a.e./liter, and they were harvested after 12 days. The boxplot for shoot dry weight shows that 3.6 × 10^−6^ g a.e./liter induces growth compared to no glyphosate, while 3.6 × 10^−3^ g a.e./liter produced toxic effects. Letters over the bars indicate significant differences (*P* < 0.05 by Tukey's honestly significant difference). Nine plates were used per treatment in two replicas. Each plate contained eight to 10 seedlings. Download FIG S1, TIF file, 0.6 MB. Copyright © 2020 Ramirez-Villacis et al. 2020 Ramirez-Villacis et al. This content is distributed under the terms of the Creative Commons Attribution 4.0 International license.

**FIG 1:**
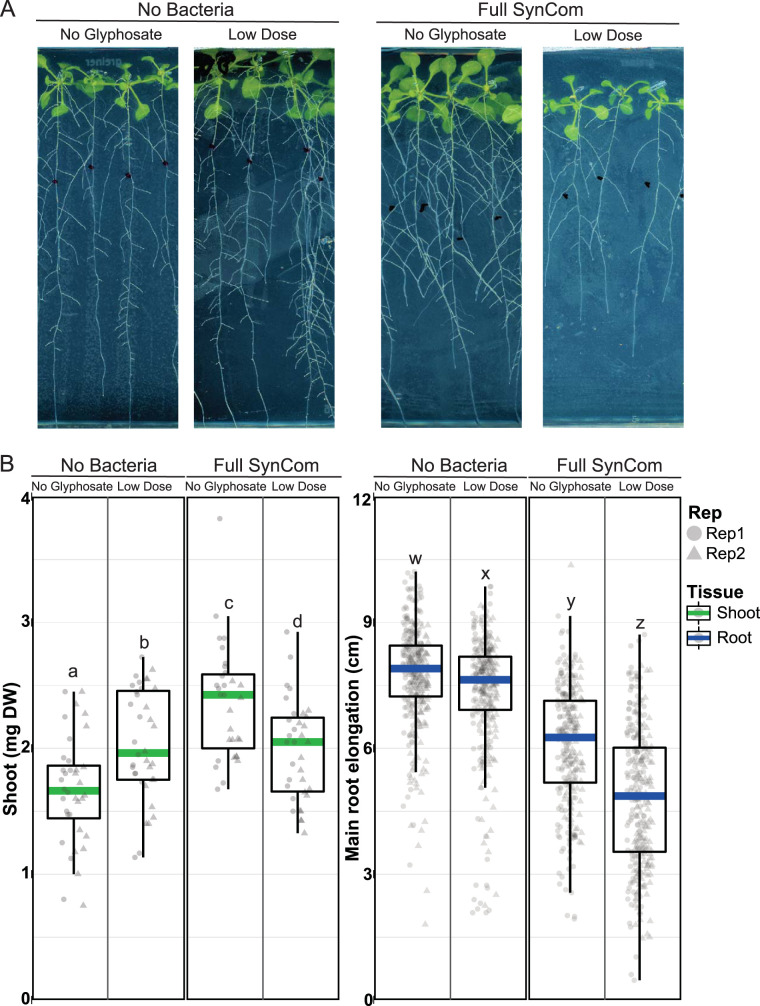
Shoot growth promotion induced by low doses of glyphosate is lost in the presence of the full bacterial synthetic community (SynCom). (A) Nineteen-day-old seedlings in half MS media with or without SynCom and a low dose of glyphosate (3.6 × 10^−6^ g a.e./liter). (B) Boxplot for shoot dry weight (DW) and main root elongation. Letters over the bars indicate significant differences (*P* < 0.05 by Tukey's honestly significant difference). Eighteen plates were used per treatment in two replicates. Each plate contained eight to 10 seedlings.

To examine the effect of the bacterial community, we applied LDG to *Arabidopsis* inoculated with the SynCom in an *in vitro* system. In accordance with previous studies, the SynCom consistently increased shoot dry weight and inhibited primary root elongation (Fig. 1A and B). These responses, however, were modulated in the presence of LDG. In the uninoculated plants, LDG produced an ∼14% increase in shoot dry weight, consistent with prior reports. This effect was lost in the presence of the SynCom. Shoot dry weight in seedlings exposed to LDG in the presence of the SynCom was reduced by ∼17% compared to the seedlings with the full SynCom and no LDG. We also noted that LDG consistently limited main root elongation, but this effect was significantly higher with the SynCom (∼2% reduction in uninoculated plants compared to ∼22% reduction with the SynCom) (Fig. 1B).

### Low doses of glyphosate produce small changes in microbiome composition of agar and root.

**FIG 2:**
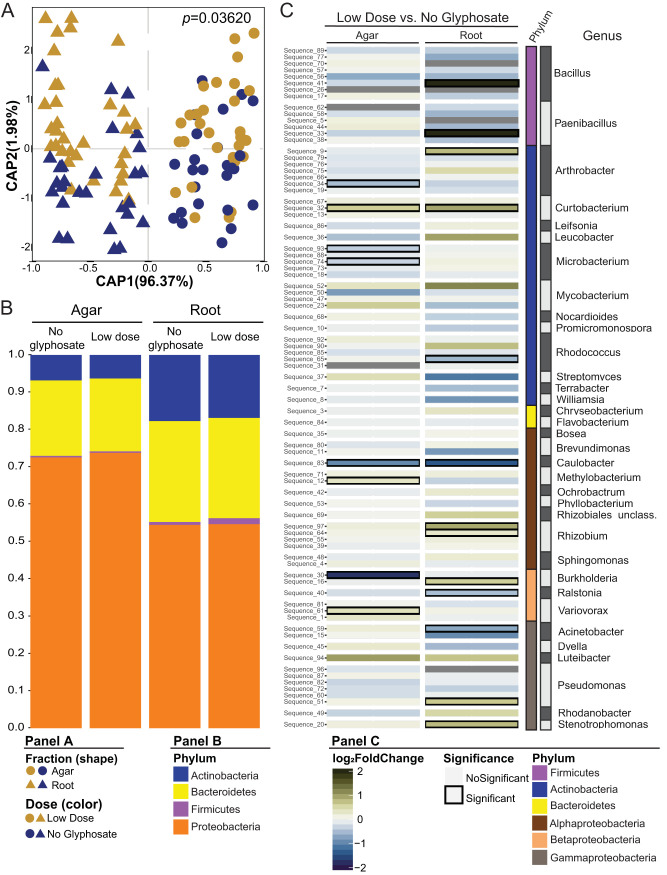
Low doses of glyphosate produce small changes in the microbiome. (A) Constrained analysis of principal coordinates (CAP) scatterplots showing the effect of the low dose of glyphosate (LDG) within agar and root fractions. *P* value from glyphosate dose, determined by PERMANOVA, is presented. (B) Bar graph representing the phylum relative abundance for each fraction and glyphosate treatment. (C) LDG enrichment patterns across the two fractions. Each row represents a unique USeq sequence from the SynCom isolates. The heatmaps are colored by log_2_ fold change. LDG enriched sequences are presented with a green gradient, while no enriched glyphosate sequences are presented with a blue gradient. Comparisons with *P* values of <0.05 are outlined in black. Eighteen plates were used per treatment in two replicas. Each plate contained eight to 10 seedlings.

The agar and root microbiomes were characterized by sequencing of the V3-V4 region of the 16S rRNA gene to assess how the SynCom was changing in response to LDG. The sequence data obtained were merged and mapped directly against full-length 16S sequences for all members of the SynCom with a 98% cutoff to identify the corresponding isolate (12). Constrained analysis of principal coordinates (CAP) showed that CAP1 separates the fractions (agar and root) and represents 96.37% of the variance, while CAP2 represents the separation between glyphosate treatments but accounts for only 1.98% of the variance (Fig. 2A). LDG induced a small yet statistically significant shift in microbiome composition (*R*^2^ = 0.00731, *P* = 0.03620). Broadly speaking, LDG slightly increased the abundance of proteobacteria in the agar while increasing the abundance of *Firmicutes* in the root. LDG depleted actinobacteria in both fractions (Fig. 2B).

To identify how specific isolates were affected by LDG, we applied a generalized linear model (15) that compared LDG versus no glyphosate within each fraction. The differentially abundant isolates were mostly found enriched in the root under the LDG condition. Two *Firmicutes* strains (one *Paenibacillus* and one *Bacillus*) were enriched in LDG by a log_2_ fold change of >2 (Fig. 2C), corresponding to a general increase in *Firmicutes* (Fig. 2B).

### Low doses of glyphosate enriched RGI strains.

**FIG 3:**
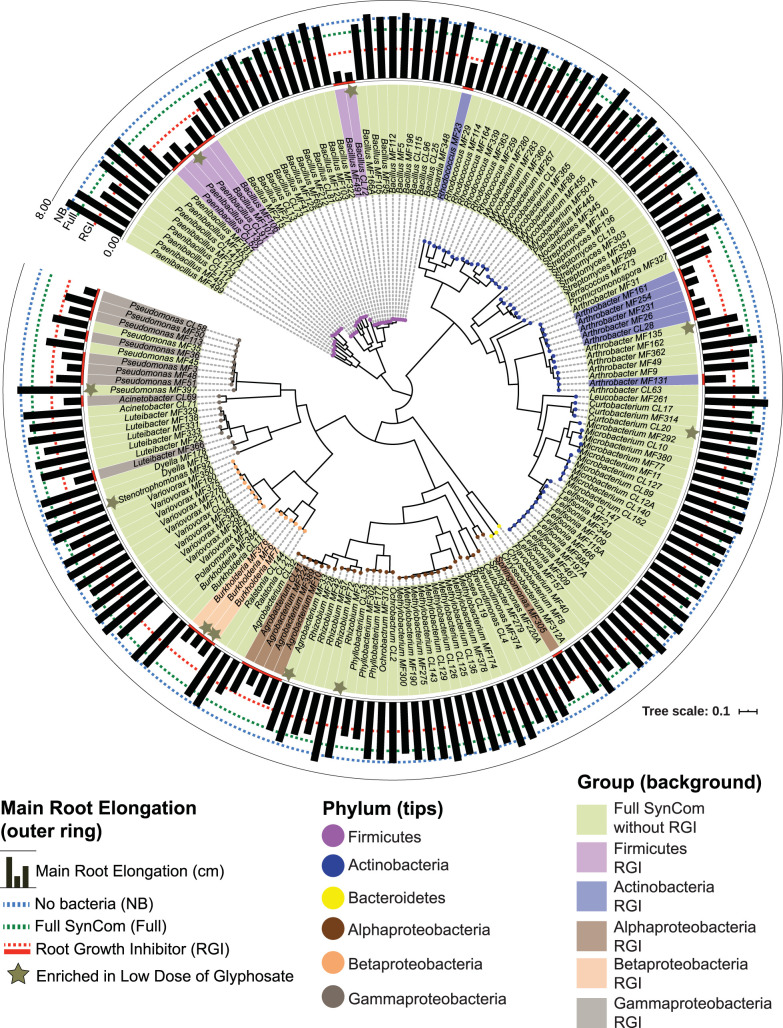
Low doses of glyphosate enriched root growth inhibitor strains. Shown is a phylogenetic tree of 185 members included in the synthetic community (SynCom), constructed using a concatenated alignment of 47 core single-copy genes. The outer ring displays the main root elongation produced by each isolate in monoassociation. NB and full correspond to the root elongation in uninoculated and SynCom treatments, respectively. A 3-cm cutoff is used to designate RGI strains (red underline). Stars mark which isolates were significantly enriched in a low dose of glyphosate in the root.

In a previous study, Finkel et al. (12) quantified the effect of each member of this SynCom on root development in a plant-bacterial strain monoassociation. Isolates that generated less than 3 cm of elongation were considered root growth inhibitors (RGI). To investigate a possible link between RGI strains and the reduction in growth produced by LDG, we compared LDG enrichment patterns to the RGI data from Finkel et al. (12) in a phylogenetic context (Fig. 3). We found that the four most highly LDG-enriched strains, *Paenibacillus* sp. strain CL91, *Bacillus* sp. strain CL72, and the two *Burkholderia* strains (MF6 and MF7), were also defined as RGI strains (Fig. 3).

### Glyphosate-induced hormesis is reversed by specific root growth-inhibiting strains within a synthetic community.

**FIG 4:**
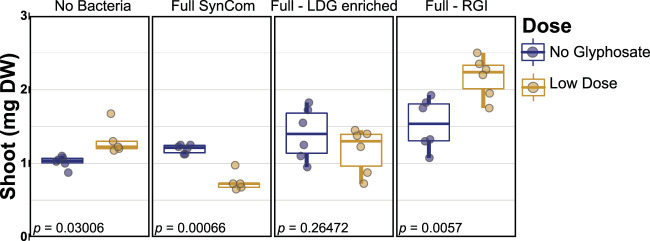
Glyphosate-induced hormesis was recovered when RGI strains were dropped out of the community. Each panel shows the effect of the low-dose glyphosate with different bacterial treatments. The second panel represents the full 185-member SynCom. The community used in the third panel does not include any of the straits that were found enriched in LDG (full-LDG enriched), while the community in the right panel does not contain any root growth inhibitor strains (full-RGI). FDR-corrected *P* values are shown within each plot. Six plates were used per treatment. Each plate contained eight to 10 seedlings.

To test the link between RGI and the growth reduction in LDG, we assembled two new synthetic communities. The first community did not include any of the 10 strains that were enriched with the LDG (marked with a star symbol in Fig. 3). The second community did not incorporate the RGI strains; thus, this new SynCom contained the 153 non-RGI isolates (green background in Fig. 3). As in previous experiments, LDG induced plant growth in the uninoculated control and reduced growth with the full SynCom. However, when the LDG-enriched strains were removed from the community (full-LDG enriched), no significant differences were observed between glyphosate treatments (Fig. 4). Moreover, when no RGI strains were incorporated (full-RGI SynCom), shoots were larger than those in the no glyphosate treatment (Fig. 4), showing growth promotion, similar to the uninoculated control, instead of growth inhibition, similar to the full SynCom.

### Individual RGI strains from across different phyla were sufficient to reduce growth in plants exposed to low doses of glyphosate.

**FIG 5:**
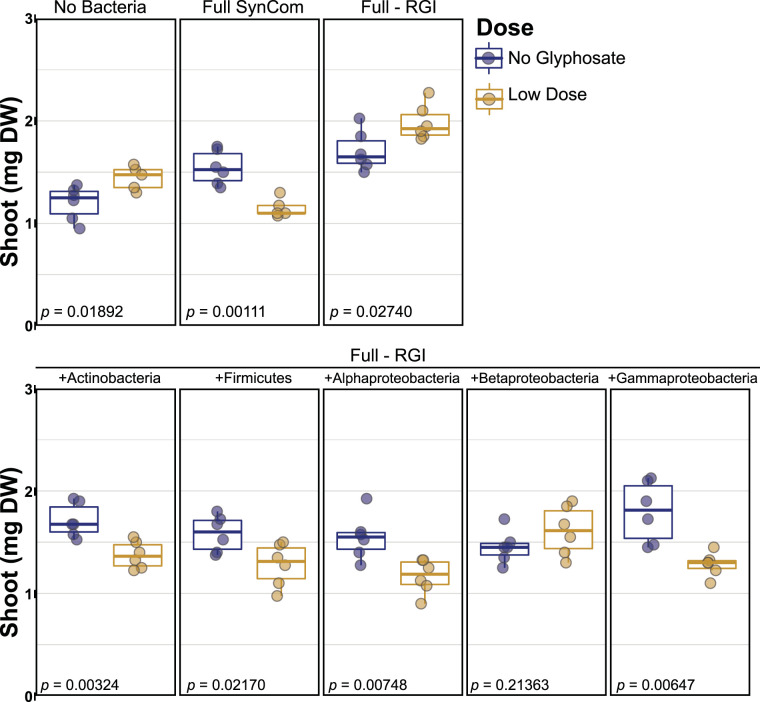
RGI strains from different phyla were sufficient to reduce growth with low doses of glyphosate. Each panel shows the effect of the low-dose glyphosate with different bacterial treatments. Top panels represent the full 185-member SynCom and the SynCom without root growth inhibitor strains (full-RGI). Bottom panels indicate the outcome after including a specific RGI group with the full-RGI community. FDR-corrected *P* values are shown within each plot. Six plates were used per treatment. Each plate contained eight to 10 seedlings.

Finally, we evaluated whether RGI strains from a specific phylum were sufficient to switch the response to LDG from growth promotion to growth inhibition. We used the SynCom without RGI (full-RGI) as a base, and then we reintroduced each specific phylogenetic group of RGI strains, as presented in Fig. 3 (grouped by color background). Any of the phylum-specific RGI isolate groups were sufficient to block growth induction and promote growth inhibition, except for betaproteobacteria (Fig. 5).

## DISCUSSION

The growth induction caused by sublethal doses of the herbicide glyphosate has long intrigued both applied and basic plant biologists. The desire to exploit hormesis for better yields in agriculture has been restricted due to inconsistent results across systems with little understanding of the underlying mechanism. Here, we readily replicated the growth promotion induced by low doses of glyphosate previously reported (2). We then investigated the role of the plant microbiome in modulating the effect of LDG using a well-characterized SynCom (12, 13).

Our data showed that low doses of glyphosate produce small changes in the composition of the root microbiome, as previously reported for higher doses (1). However, even with the lack of major shifts in the community, there was a clear effect over the phenotype. Our results indicate that glyphosate hormesis is entirely dependent on the root microbiome composition, especially on the presence/absence of root growth inhibitors. Given that RGI strains are scattered across the bacterial phylogeny and could be found in natural root microbiomes, their enrichment under low doses of glyphosate could account for the reduced number of examples of glyphosate hormesis in the field. Further, the broad presence of RGI likely limits the utility of applying low doses of glyphosate as a growth promoter in agricultural or wild settings.

While RGI isolates within the plant-associated microbiome shape part of a plant’s response to glyphosate, the interplay is more complex. When LDG-enriched strains were removed from the community, hormesis was not recovered, pointing out that other nonenriched strains also play an important role in response to LDG. Interestingly, although LDG enrichment patterns were informative for discovering the link between LDG and RGI, these two factors were not concordant across the phylogeny. In fact, only in the case of *Firmicutes* did we observed RGI, LDG enrichment, and hormesis reversal. We observed that RGI strains from other phyla that are also capable of reversing hormesis were not LDG enriched. This could be explained by a competitive model where *Firmicutes* strains have an advantage for exploiting the chemical changes caused by LDG.

Assembly of the root microbiome depends in part on root exudates, specifically on aromatic organic acids and amino acids (16, 17). At recommended doses, glyphosate inhibits growth by blocking the activity of the EPSPS enzyme, resulting in the reduction of cyclic amino acid production (1). Consistent with this idea, overexpressing exogenous EPSPS in *Arabidopsis*, in the absence of glyphosate, results in a dry mass gain and higher auxin content (18). In soybean exposed to low doses, plants excreted higher levels of cyclic amino acids and other aromatic compounds downstream of EPSPS. Tryptophan, in particular, increased by 80% in concentration relative to the control (19). Thus, if LDG alters the production of cyclic compounds and tryptophan (19), then this change could account for the variations in the microbiome and, perhaps, the recruitment of RGI strains. These RGI strains then could alter the plant’s root morphology, perhaps due to their increased abundance. Consistent with this point, the growth increase observed by overexpression of EPSPS has been observed *in vitro* or in greenhouse experiments for various species, but contradictory results were found in field experiments (20). This also suggests that the presence of RGI bacteria in the soil microbiome modulates this effect on shoot growth.

Bacterial root growth inhibition could arise from different mechanisms, such as the production of toxic compounds (21), presence of pathogen-associated-molecular-pattern (PAMP) or microbe-associated molecular pattern (MAMP) (22, 23), and production of auxin and auxin-like compounds (12). These mechanisms are widespread across bacterial phyla, and one strain could harbor more than one (12, 21–23). Various isolates from the 185-member SynCom activate auxin- and ethylene-related pathways (12). Tryptophan-dependent IAA synthesis pathways were found in 82.2% of 7,282 bacterial genomes associated with plant root environments (24). Increased tryptophan concentration in the root and root vicinity is related to enhancing the production of auxin/auxin-like compounds by different bacteria, which could result in enhanced growth inhibition (25–28). This could explain why it was necessary to remove all root growth inhibitor isolates from the community to completely recover LDG hormesis.

In sum, using a top-down approach, we assembled different synthetic communities that modulated the plant response to low doses of glyphosate, from growth inhibition to growth promotion, depending on the presence of root growth inhibitors. Our work suggests connections between LDG, increased expression of EPSPS, and growth promotion. However, establishing this link requires further transcriptomic and metabolomic analysis as well as complementary experiments linking tryptophan overproduction induced by low doses of glyphosate and growth-inhibiting microorganisms. Even further, our study hints at the significance of the root microbiome structure in plant responses to abiotic factors with enormous implications far beyond the topic of the current study.

## MATERIALS AND METHODS

### *In vitro* plant growth conditions.

Arabidopsis thaliana Columbia 0 (Col-0) seeds were surface sterilized for 10 min with 70% bleach plus 0.2% Tween 20, rinsed three times with sterile distilled water, and stratified at 4°C for 2 days in the dark. Between 40 to 50 seeds were sowed on vertical square plates with half-strength MS supplemented with 0.5 g/liter morpholineethanesulfonic acid (MES), 5 g/liter sucrose, 10 g/liter Bacto-agar (Difco, BD, Franklin Lakes, NJ, USA) with a final pH of 5.6 to 5.7. After 7 days, 10 seedlings were transferred to vertical square plates with half-strength MS supplemented with 0.5 g/liter MES and 10 g/liter Bacto-agar, with or without the synthetic community (SynCom) and the different glyphosate treatments with a final pH of 5.6 to 5.7. For the dose standardization experiment, 3.6 × 10^−3^ and 3.6 × 10^−6^ g a.e./liter were assessed; for the rest of the experiments, 3.6 × 10^−6^ g a.e./liter was used as a low dose of glyphosate (LDG). Each experiment included a no glyphosate control treatment and an uninoculated control. Plates were set randomly in a growth chamber with a 16-h dark/8-h light regimen at 21°C day/18°C night for 12 days.

### Bacterial culture and plant inoculation.

The bacterial isolates used were previously obtained and sequenced in a previous study by Levy et al. (29). Seven days before each experiment, glycerol stocks from each isolate were inoculated in 400 μl KB medium in a 96-deep-well plate. The plates were incubated at 28°C at 250 rpm. After 5 days, 40 μl of the liquid culture was transferred to a new 96-well plate with fresh 400 μl KB medium and grown under the same conditions for 2 days. Ultimately, 7-day and 2-day liquid cultures were combined for each isolate. This procedure accounts for variable growth rates and aims to ensure that nonstationary cells are present in the final inoculum, as previously described by Finkel et al. (12). The number of cells for each isolate was normalized to an optical density at 600 nm (OD_600_) equal to 1 (Infinite M200 Pro plate reader; TECAN, Männedorf, Switzerland) in the final pool. The mixed culture was washed three times with 10 mM MgCl_2_ to remove the media and debris. The washed mixed culture then was diluted to a final OD_600_ of 0.2, and 100 μl of the inoculum was spread on 12- by 12-cm vertical square agar plates with the corresponding medium before transfer of the seedlings.

### DNA extraction.

Roots were pooled from 6 to 8 plants for each plate and placed in 2.0-ml Eppendorf tubes with three sterile glass beads. The samples then were washed three times with sterile distilled water and frozen with liquid nitrogen. Roots were lyophilized for 48 h (Labconco freeze-dry system, Kansas City, MO, USA) and pulverized (tissue homogenizer; MPBio, Munich, Germany). Agar from each plate was collected in 60-ml syringes with sterilized Miracloth (Millipore, Burlington, MA, USA) at the tip and stored at –20°C. After 1 week, syringes were thawed at room temperature and then gently pressed through the Miracloth into 50-ml tubes. The liquid samples were centrifuged at 4,200 rpm for 20 min, and the supernatant was discarded. The remaining liquid with the pellet was transferred into a 2.0-ml Eppendorf tube and centrifuged, all supernatant was removed, and the pellet was stored at –80°C. DNA from root samples and pellets from agar were carried out using a 96-well format DNeasy PowerSoil kit (Qiagen, Hilden, Germany) by following the manufacturer’s instructions. Samples were randomized in the plates and maintained throughout library preparation and sequencing.

### Library preparation and sequencing.

Library preparation was done according to reference 30, using a dual-index approach. The V3-V4 region of the bacterial 16S rRNA gene was amplified using the primers 338F (5′-ACTCCTACGGGAGGCAGCA-3′) and 806R (5′-GGACTACHVGGGTWTCTAAT-3′). PCR was performed with the following components: 1.5 μl of 10 μM each primer, 1 μl of 10 μM mitochondrial PNA, 1 μl of 10 μM plastid PNA, 6.5 μl PCR-grade water, 12.5 μl Kappa master mix (Roche, Indianapolis, IN, USA), and 1 μl gDNA template. Temperature cycling was 3 min at 95°C, followed by 20 cycles of 15 s at 95°C, 15 s at 78°C, 15 s at 50°C, and 15 s at 72°C. PCRs were done in triplicate, and amplification was checked on 1.5% agarose gels at 100 V for 35 min. The triplicate reactions were pooled and purified using AMPure XP magnetic beads (Beckman Coulter, High Wycombe, UK) and quantified with a Qubit BR DNA assay (Invitrogen, Carlsbad, CA, USA). Libraries were pooled in equal amounts and then diluted to 10 pM for sequencing on an Illumina MiSeq instrument (Illumina, San Diego, CA, USA) using a 600-cycle V3 chemistry kit.

### Amplicon sequence data processing.

Reads with 100% correct primer sequences were merged (MT-Toolbox) (31) and quality filtered (Sickle) (32) for a Q score of >20. The merge sequences were globally aligned to the 16S rRNA gene sequences of the 185 isolates in the SynCom (USEARCH v7.1090) (33) and were classified into 97 unique sequences (USeq). A USeq is a cluster of 100% identical sequences coming from a single or multiples isolates, as previously done by Finkel et al. (12). Match between USeq sequences and strain identities can be found in Table S1 in the supplemental material. On average, 80% of sequences were assigned to an expected USeq. The unmapped sequences were clustered into operational taxonomic units (OTUs) using UPARSE (34) at 97% identity. Representative OTU sequences were taxonomically annotated with the RDP classifier (35) trained on the Greengenes database (36) (4 February 2011). *Arabidopsis* organellar and known bacterial contaminants were removed using the option “usearch_global” at a 98% identity threshold (USEARCH v7.1090) (33). USeq mapped sequences and OTU counts were used to produce a combined abundance table. The table was processed and analyzed with functions from the Ohchibi package (https://github.com/isaisg/ohchibi).

10.1128/mSphere.00484-20.2 TABLE S1 USeq sequence identification and taxonomy for each strain in the 185-member SynCom. Download Table S1, CSV file, 0.02 MB. Copyright © 2020 Ramirez-Villacis et al. 2020 Ramirez-Villacis et al. This content is distributed under the terms of the Creative Commons Attribution 4.0 International license.

The resulting count table was rarefied to 1,000 reads per sample. Beta diversity was analyzed with a canonical analysis of principal coordinates (CAP) based on Bray-Curtis dissimilarity calculated from the relative abundance matrices. Dose interaction analysis was performed with constraining for the replica effect. A permutational multivariate analysis of variance (PERMANOVA) also was performed using the adonis function from vegan package v2.5-3 (37).

To establish the enrichment profiles in the comparison of LDG to no glyphosate from each fraction, we employed the package DESeq2 v1.22.1 (38) to run the model abundance ∼ dose + repetition using the raw USeq/OTU combined count table. A USeq/OTU was considered statistically significant if it had a false discovery rate (FDR)-adjusted *P* value of <0.05.

### Phylogenetic tree.

The phylogenetic tree of the SynCom isolates was previously constructed by Finkel et al. (12). We selected the same 47 markers and the same approach to create a superalignment and to infer the phylogeny utilizing the WAG model of evolution (FastTree v2.1) (39). We then used a web-based tool (https://itol.embl.de/) to visualize the tree and to add information on main root elongation from each isolate in monoassociation, available in Data Set S4 (https://www.biorxiv.org/content/10.1101/645655v1.supplementary-material) from reference 12.

### Growth assessment.

Shoot and root growth were measure 12 days after transferring to the specific media, as described in “*In vitro* plant growth conditions,” above. For main root elongation, plates were imaged using a document scanner, and the primary root length from each plant was measured using ImageJ. Shoots were harvested for dry weight. Six to eight shoots from one plate were put in a preweighed 2.0-ml Eppendorf tube and placed in an oven at 60°C for 72 h, when the weight of the tubes was stable. To calculate the dry weight, the initial weight of the tube was subtracted from the weight of the tube with the shoots after 72 h and divided by the number of shoots placed in each tube. For the experiment presented in Fig. 1, main root elongation and shoot dry weight were assessed, while for dose standardization (Fig. S1) and dropout experiments (Fig. 3 and 4), only shoot dry weight was used.

### Statistical analysis.

Analyses of variance (ANOVA), controlling for the replicate effect, was used for Fig. 1B and Fig. S1. Differences between treatments were shown using the confidence letter display (CLD) derived from Tukey’s *post hoc* test (package emmeans) (40).

Statistical analysis used in Fig. 2 is explained in “Amplicon sequence data processing,” above. For Fig. 3 and 4, differences between no glyphosate and low dose were analyzed using Student's *t* test, adjusting the *P* values for multiple testing using FDR, performed in the R package (41, 42). The number of replicates is given in the respective figure legends.

### Data availability.

Amplicon sequencing data are available at the NCBI Sequence Read Archive (project PRJNA632632). Count tables and relevant data files can be found at GitHub (https://github.com/darioxr/glyphosate_syncom).

## References

[1] Duke SO, Lydon J, Koskinen WC, Moorman TB, Chaney RL, Hammerschmidt R. 2012. Glyphosate effects on plant mineral nutrition, crop rhizosphere microbiota, and plant disease in glyphosate-resistant crops. J Agric Food Chem 60:10375–10397. doi:10.1021/jf302436u. 23013354 PMC3479986

[2] Brito IP, Tropaldi L, Carbonari CA, Velini ED. 2018. Hormetic effects of glyphosate on plants. Pest Manag Sci 74:1064–1070. doi:10.1002/ps.4523. 28094904

[3] Velini ED, Alves E, Godoy MC, Meschede DK, Souza RT, Duke SO. 2008. Glyphosate applied at low doses can stimulate plant growth. Pest Manag Sci 64:489–496. doi:10.1002/ps.1562. 18293284

[4] Ather Nadeem M, Abbas T, Tanveer A, Maqbool R, Zohaib A, Shehzad MA. 2017. Glyphosate hormesis in broad-leaved weeds: a challenge for weed management. Arch Agron Soil Sci 63:344–351. doi:10.1080/03650340.2016.1207243.

[5] Wagner R, Kogan M, Parada AM. 2003. Phytotoxic activity of root absorbed glyphosate in corn seedlings (Zea mays L.). Weed Biol Manage 3:228–232. doi:10.1046/j.1444-6162.2003.00110.x.

[6] De Carvalho LB, Alves PLCA, Duke SO. 2013. Hormesis with glyphosate depends on coffee growth stage. An Acad Bras Cienc 85:813–822. doi:10.1590/S0001-37652013005000027. 23828346

[7] Pokhrel LR, Karsai I. 2015. Long-term sub-lethal effects of low concentration commercial herbicide (glyphosate/pelargonic acid) formulation in Bryophyllum pinnatum. Sci Total Environ 538:279–287. doi:10.1016/j.scitotenv.2015.08.052. 26311583

[8] Cedergreen N, Felby C, Porter JR, Streibig JC. 2009. Chemical stress can increase crop yield. Field Crops Res 114:54–57. doi:10.1016/j.fcr.2009.07.003.

[9] El-Shahawy TA, Sharara FA. 2011. Hormesis influence of glyphosate in between increasing growth, yield and controlling weeds in faba bean. J Am Sci 7:139–144.

[10] Fierer N. 2017. Embracing the unknown: disentangling the complexities of the soil microbiome. Nat Rev Microbiol 15:579–590. doi:10.1038/nrmicro.2017.87. 28824177

[11] Newman MM, Lorenz N, Hoilett N, Lee NR, Dick RP, Liles MR, Ramsier C, Kloepper JW. 2016. Changes in rhizosphere bacterial gene expression following glyphosate treatment. Sci Total Environ 553:32–41. doi:10.1016/j.scitotenv.2016.02.078. 26901800

[12] Finkel OM, Salas-Gonzalez I, Castrillo G, Conway JM, Law TF, Teixeira PJPL, Wilson ED, Fitzpatrick CR, Jones CD, Dangl JL. 2020. A single bacterial genus maintains root development in a complex microbiome. bioRxiv doi:10.1101/645655. PMC10329457 32999461

[13] Finkel OM, Salas-González I, Castrillo G, Spaepen S, Law TF, Teixeira PJPL, Jones CD, Dangl JL. 2019. The effects of soil phosphorus content on plant microbiota are driven by the plant phosphate starvation response. PLoS Biol 17:e3000534. doi:10.1371/journal.pbio.3000534. 31721759 PMC6876890

[14] Tsolakidou MD, Stringlis IA, Fanega-Sleziak N, Papageorgiou S, Tsalakou A, Pantelides IS. 2019. Rhizosphere-enriched microbes as a pool to design synthetic communities for reproducible beneficial outputs. FEMS Microbiol Ecol 95:fiz138. doi:10.1093/femsec/fiz138. 31504462

[15] Anders S, Huber W. 2010. Differential expression analysis for sequence count data. Genome Biol 11:R106. doi:10.1186/gb-2010-11-10-r106. 20979621 PMC3218662

[16] Zhalnina K, Louie KB, Hao Z, Mansoori N, da Rocha UN, Shi S, Cho H, Karaoz U, Loqué D, Bowen BP, Firestone MK, Northen TR, Brodie EL. 2018. Dynamic root exudate chemistry and microbial substrate preferences drive patterns in rhizosphere microbial community assembly. Nat Microbiol 3:470–480. doi:10.1038/s41564-018-0129-3. 29556109

[17] Sasse J, Martinoia E, Northen T. 2018. Feed your friends: do plant exudates shape the root microbiome? Trends Plant Sci 23:25–41. doi:10.1016/j.tplants.2017.09.003. 29050989

[18] Fang J, Nan P, Gu Z, Ge X, Feng YQ, Lu BR. 2018. Overexpressing exogenous 5-enolpyruvylshikimate-3-phosphate synthase (EPSPS) genes increases fecundity and auxin content of transgenic arabidopsis plants. Front Plant Sci 9:233. doi:10.3389/fpls.2018.00233. 29535747 PMC5835131

[19] Silva FML, Duke SO, Dayan FE, Velini ED. 2016. Low doses of glyphosate change the responses of soyabean to subsequent glyphosate treatments. Weed Res 56:124–136. doi:10.1111/wre.12189.

[20] Yang X, Beres ZT, Jin L, Parrish JT, Zhao W, Mackey D, Snow AA. 2017. Effects of over-expressing a native gene encoding 5-enolpyruvylshikimate-3-phosphate synthase (EPSPS) on glyphosate resistance in Arabidopsis thaliana. PLoS One 12:e0175820. doi:10.1371/journal.pone.0175820. 28426703 PMC5398549

[21] Hogenhout SA, Loria R. 2008. Virulence mechanisms of Gram-positive plant pathogenic bacteria. Curr Opin Plant Biol 11:449–456. doi:10.1016/j.pbi.2008.05.007. 18639483

[22] Teixeira PJP, Colaianni NR, Fitzpatrick CR, Dangl JL. 2019. Beyond pathogens: microbiota interactions with the plant immune system. Curr Opin Microbiol 49:7–17. doi:10.1016/j.mib.2019.08.003. 31563068

[23] Pel MJ, Pieterse CM. 2013. Microbial recognition and evasion of host immunity. J Exp Bot 64:1237–1248. doi:10.1093/jxb/ers262. 23095994

[24] Zhang P, Jin T, Kumar Sahu S, Xu J, Shi Q, Liu H, Wang Y. 2019. The distribution of tryptophan-dependent indole-3-acetic acid synthesis pathways in bacteria unraveled by large-scale genomic analysis. Molecules 24:1411. doi:10.3390/molecules24071411. PMC6479905 30974826

[25] Jaeger CH, Lindow SE, Miller W, Clark E, Firestone MK. 1999. Mapping of sugar and amino acid availability in soil around roots with bacterial sensors of sucrose and tryptophan. Appl Environ Microbiol 65:2685–2690. doi:10.1128/AEM.65.6.2685-2690.1999. 10347061 PMC91396

[26] Sarwar M, Kremer RJ. 1995. Enhanced suppression of plant growth through production of L-tryptophan-derived compounds by deleterious rhizobacteria. Plant Soil 172:261–269. doi:10.1007/BF00011328.

[27] Kravchenko LV, Azarova TS, Makarova NM, Tikhonovich IA. 2004. The effect of tryptophan present in plant root exudates on the phytostimulating activity of rhizobacteria. Microbiology 73:156–158. doi:10.1023/B:MICI.0000023982.76684.9d. 15198030

[28] Barazani OZ, Friedman J. 2000. Effect of exogenously applied L-tryptophan on allelochemical activity of plant-growth-promoting rhizobacteria (PGPR). J Chem Ecol 26:343–349. doi:10.1023/A:1005449119884.

[29] Levy A, Salas Gonzalez I, Mittelviefhaus M, Clingenpeel S, Herrera Paredes S, Miao J, Wang K, Devescovi G, Stillman K, Monteiro F, Rangel Alvarez B, Lundberg DS, Lu T-Y, Lebeis S, Jin Z, McDonald M, Klein AP, Feltcher ME, Rio TG, Grant SR, Doty SL, Ley RE, Zhao B, Venturi V, Pelletier DA, Vorholt JA, Tringe SG, Woyke T, Dangl JL. 2018. Genomic features of bacterial adaptation to plants. Nat Genet 50:138–150. doi:10.1038/s41588-017-0012-9. PMC5957079 29255260

[30] Gohl DM, Vangay P, Garbe J, MacLean A, Hauge A, Becker A, Gould TJ, Clayton JB, Johnson TJ, Hunter R, Knights D, Beckman KB. 2016. Systematic improvement of amplicon marker gene methods for increased accuracy in microbiome studies. Nat Biotechnol 34:942–949. doi:10.1038/nbt.3601. 27454739

[31] Yourstone SM, Lundberg DS, Dangl JL, Jones CD. 2014. MT-Toolbox: improved amplicon sequencing using molecule tags. BMC Bioinformatics 15:284. doi:10.1186/1471-2105-15-284. 25149069 PMC4153912

[32] Joshi NA, Fass JN. 2011. Sickle: a sliding-window, adaptive, quality-based trimming tool for FastQ files (version 1.33). https://github.com/najoshi/sickle.

[33] Edgar RC. 2010. Search and clustering orders of magnitude faster than BLAST. Bioinformatics 26:2460–2461. doi:10.1093/bioinformatics/btq461. 20709691

[34] Edgar RC. 2013. UPARSE: highly accurate OTU sequences from microbial amplicon reads. Nat Methods 10:996–998. doi:10.1038/nmeth.2604. 23955772

[35] Wang Q, Garrity GM, Tiedje JM, Cole JR. 2007. Naive Bayesian classifier for rapid assignment of rRNA sequences into the new bacterial taxonomy. Appl Environ Microbiol 73:5261–5267. doi:10.1128/AEM.00062-07. 17586664 PMC1950982

[36] DeSantis TZ, Hugenholtz P, Larsen N, Rojas M, Brodie EL, Keller K, Huber T, Dalevi D, Hu P, Andersen GL. 2006. Greengenes, a chimera-checked 16S rRNA gene database and workbench compatible with ARB. Appl Environ Microbiol 72:5069–5072. doi:10.1128/AEM.03006-05. 16820507 PMC1489311

[37] Oksanen J. 2019. Package “vegan.” https://cran.r-project.org, https://github.com/vegandevs/vegan.

[38] Love MI, Huber W, Anders S. 2014. Moderated estimation of fold change and dispersion for RNA-seq data with DESeq2. Genome Biol 15:550. doi:10.1186/s13059-014-0550-8. 25516281 PMC4302049

[39] Price MN, Dehal PS, Arkin AP. 2010. FastTree 2–approximately maximum-likelihood trees for large alignments. PLoS One 5:e9490. doi:10.1371/journal.pone.0009490. 20224823 PMC2835736

[40] Lenth RV. 2019. R package “emmeans”: estimated marginal means. https://github.com/rvlenth/emmeans.

[41] R Core Development Team. 2013. R: a language and environment for statistical computing. R Foundation for Statistical Computing, Vienna, Austria.

[42] RStudio Team. 2012. RStudio: integrated development environment for R. PBC, Boston, MA.

