# Supplementary figures and images for: Root Microbiome Modulates Plant Growth Promotion Induced by Low Doses of Glyphosate
Source: mSphere. 2020 Aug 12;5(4):e00484-20. doi: 10.1128/mSphere.00484-20 (PMC7426167; doi:10.1128/mSphere.00484-20)

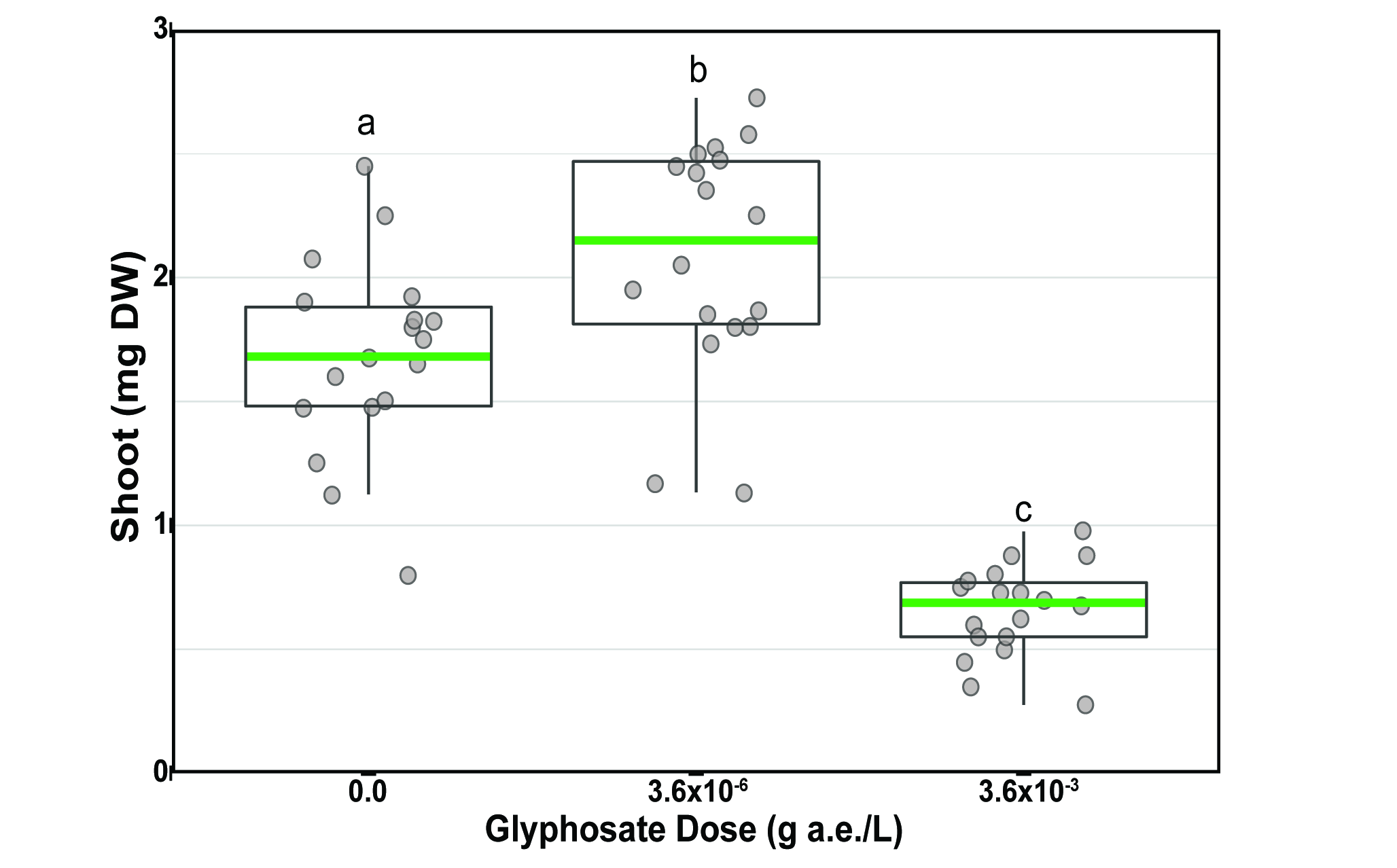

Supplement: FIG S1 [file mSphere.00484-20-sf001.tif]
